# Patterns of help-seeking behavior among people with mental illness in Ethiopia: a systematic review and meta-analysis

**DOI:** 10.3389/fpsyt.2024.1361092

**Published:** 2024-03-18

**Authors:** Sintayehu Asnakew, Kalkidan Haile, Bekalu Getnet Kassa, Gashaw Wale Ayehu, Getnet Mihretie Beyene, Dejen Getaneh Feleke, Desalegn Gizachew Endalew, Getasew Legas, Birhanu Mengist Munie, Assasahegn Tedila, Kirubel Shiferaw, Amsalu Belete, Ermias Sisay Chanie, Tigabu Munye Aytenew

**Affiliations:** ^1^ Department of Psychiatry, College of Health Science, Debre Tabor University, Debre Tabor, Ethiopia; ^2^ Department of Psychiatry, Amhara Public Health Institute, Bahirdar, Ethiopia; ^3^ Department of Midwifery, College of Health Sciences, Debre Tabor University, Debre Tabor, Ethiopia; ^4^ Department of Anatomy, College of Health Sciences, Debre Tabor University, Debre Tabor, Ethiopia; ^5^ Department of Pediatrics and Child Health Nursing, College of Health Sciences, Debre Tabor University, Debre Tabor, Ethiopia; ^6^ Department of Psychiatry, Debre Tabor Comprehensive Specialized Hospital, Debre Tabor, Ethiopia; ^7^ Department of Nursing, College of Health Sciences, Debre Tabor University, Debre Tabor, Ethiopia

**Keywords:** mental illness, Ethiopia, help seeking behavior, systematic review and meta-analysis, treatment

## Abstract

**Background:**

Despite the availability of evidence-based and effective treatments, significant numbers of people living with mental illness do not receive treatment or do not seek help from providers of formal modern treatment. Although numerous primary studies have been conducted on patterns of help-seeking behavior among individuals with mental illness with respect to modern therapy, the evidence has not been aggregated nationwide. Therefore, the aim of this review was to investigate pooled data on patterns of help-seeking behavior among individuals with mental illness in Ethiopia.

**Methods:**

All available primary studies were searched via the Google Scholar, HINARI, and PubMed databases from June 22 to December 20, 2023; 912 articles were identified. Sixteen articles were included in the final review; data from them were extracted to an Excel spreadsheet and exported to Stata version 17 for analysis. The search terms used were: “Pattern of help-seeking behavior’’ OR “Pattern of treatment-seeking behavior” OR “Health care-seeking behavior” OR “Help-seeking intention” OR “Help-seeking preferences” OR “Perceived need” OR “Pathways to psychiatric care”, AND “Common mental disorders” OR “Mental illness” OR “Mental health problems” OR “Depression”, AND “Predictors” OR “Determinate factors” OR “Associated factors”, AND “Ethiopia”. The quality of the studies included was critically appraised using the modified The Joanna Briggs Institute (JBI) Joanna Briggs Institute quality assessment tool, adapted for observational studies. During critical appraisal, disagreements between the two authors conducting the assessment were resolved by the involvement of a third author. Effect sizes were pooled using the random effects model, and the presence of publication bias was detected based on asymmetry of the funnel plot and a statistically significant result of Egger’s test (p<0.05).

**Results:**

The pooled rate of positive help-seeking behavior with respect to modern treatment among people living with mental illness was 42.21% (95% CI: 29.29, 55.12; I^2^ = 99.37%, P=0.00). Factors significantly associated with a positive pattern of help-seeking behavior were: having a secondary education or above (AOR=5.47, 95% CI: 2.33, 12.86); believing that mental illness requires treatment (AOR=2.76, 95% CI: 2.02, 3.78); having strong social support (AOR=2.00, 95% CI: 1.64, 2.44); having a family history of mental illness (AOR=2.68, 95% CI: 1.38, 3.97); having awareness of the availability of treatment (AOR=2.92, 95% CI: 1.56, 5.46); having previously engaged in positive help-seeking behavior (AOR=3.28, 95% CI: 1.63, 6.60); having comorbid disorders (AOR=4.25, 95% CI: 1.69, 10.66); not using alcohol (AOR=3.29, 95% CI: 1.73, 6.27); and the perceived severity of mental illness (AOR=2.54, 95% CI: 1.490, 4.33).

**Conclusions:**

The majority of people with mental illness in Ethiopia exhibited a poor pattern of help-seeking behavior with respect to modern treatment. Therefore, mobilization of the community should be encouraged via regular public awareness campaigns regarding mental illness and the availability of evidence-based and effective modern treatment in Ethiopia. Moreover, the design of effective community-based mental health interventions is recommended in order to improve public attitudes and rates of help-seeking behavior in relation to mental health problems.

## Introduction

The World Health Organization (WHO) defines mental health as a subjective state of well-being in which a person recognizes their own potential and is able to manage the typical stressors of everyday life, work productively, and contribute to their community (1).

Patients’ cultural beliefs about the causes and experience of mental illness affect their attitudes toward effective treatment, as well as the type of treatment they seek. In Ethiopia, mentally ill individuals are frequently blamed for their own condition; alternatively, some may consider mentally ill people to be victims of bad luck, religious and moral transgression, and witchcraft (2–4). This may result in denial on the part of both patients and their families, causing delays in seeking expert treatment.

Myths and beliefs about mental health and sickness exist in every community, and they impact people’s attitudes (5). Attitudes toward mental illnesses and their treatment among the majority of African societies, including Ethiopian society, differ from scientific views, which may have a negative impact on treatment-seeking behavior (6).

Mental illness is among the most poorly recognized health issues in Ethiopia. Societal prejudice and stigma against mental illness jeopardize the delivery of high-quality comprehensive patient care and rehabilitation (7, 8). Furthermore, negative views in society hinder mentally ill people from seeking and adhering to treatment (9, 10).

Mental illnesses and related disorders accounted for 13% of all diseases worldwide in 2019 (11), and this figure is expected to raise to 15% by 2020. Moreover, behavioral disorders are linked to significant rates of disability in low- and middle-income countries (LMICs). In LMICs, these conditions contribute 25.3% and 33.5%, respectively, of the total years for which a person lives with a disability (12).

WHO reports indicate that 41% of patients who attend the outpatient department of a psychiatric hospital are diagnosed with schizophrenia, 13% with mood disorders, and 11% with other disorders. Among patients who receive inpatient care, 85% are diagnosed with mood disorders or schizophrenia (13).

Worldwide, the mental health treatment gap is significant. Treatment for severe mental health problems is never received by up to 85% of patients in low-income countries and 35–50% of patients in high-income countries (14). Other research also indicates that approximately 70% of people globally who suffer from mental illness never receive treatment from mental health professionals (15). Despite the fact that mental health disorders are widespread and severely debilitating for people worldwide, there is limited demand from society for the services provided by modern facilities (16).

It is evident that the rates of help-seeking behavior among people with mental illness with respect to modern treatment in various regions of the world are low. In Rwanda, only 36.0% of respondents to a survey were found to seek help at a modern facility (17); this figure has been found to be 28% in England (18), 28.2% in China (19), 28.4% in Brazil (20), 32% in Singapore (21), 46.2% in Indonesia (22), 52% in another Chinese study (23), 51.1% in another Brazilian study (24), 65% in the Netherlands (25), 58.9% in Zanzibar (26), 64.6% in Hunan, China (27), 85% in Australia (28), 90.3% in Egypt (29), and 12.7% in another Chinese study (30). Furthermore, an Indian study revealed that, while a comparatively large number of respondents (35%) preferred traditional healers as providers of treatment for their mental health issues, fewer respondents (18%) said that they may see a psychiatrist if they were experiencing emotional problems (9). Another study carried out in Nigeria revealed that, in terms of patterns of help-seeking, 46% of respondents preferred traditional medical care as their primary source of assistance, 34% preferred spiritual healing, and 18% preferred informal help over modern medicine (31).

In Ethiopia, mental illness is the most common form of non-infectious disease, accounting for up to 11% of all illnesses, which is higher than the burden resulting from HIV/AIDS (32). However, the treatment gap for mental health issues is significant. Most people do not seek help or delay seeking help, which has serious consequences in the form of personal, social, and economic costs (33, 34). Some patients have reported being unsure of where to obtain help, and 20.1% say that they do not want others to know about their problems (35). The community may also transfer patients with mental health problems to modern medical services when they have lost hope, with no progress made after exhausting informal traditional methods, such as herbal treatments and holy water (16, 36). Furthermore, in certain regions of Ethiopia, individuals may abandon mentally ill patients if they do not respond to modern therapies and informal source of help. Lastly, some patients may walk the streets in their underwear, or live with family and receive their basic necessities in this way (37).

Generally, people with mental illnesses in Ethiopia show poor rates of engaging in help-seeking behavior with respect to modern health services, with rates ranging from 16.2 to 81.5% (4, 38–52). Although several primary studies have been conducted in different areas to examine this problem, there are major discrepancies in the findings, with inconsistent results having been obtained across various regions within Ethiopia. Therefore, the aim of this systematic review and meta-analysis was to synthesize the pooled evidence on patterns of positive help-seeking behavior among people with mental illness with respect to modern treatment, and predictors of this behavior, in Ethiopia. Thus, the current study will provide information for policymakers and concerned stakeholders for the design of prevention strategies and strategies to increase the help-seeking behavior of people with mental illness with respect to modern treatment. Furthermore, the findings of this review can be used as an input for researchers who intend to conduct further investigations in this area.

## Methods

### Reporting and registration protocol

The Preferred Reporting Items for Systematic Reviews and Meta-Analyses (PRISMA) statement guidelines (53) were followed in reporting the results of this systematic review and meta-analysis (**Supplementary Table 1**). The review protocol was registered with the Prospero database (PROSPERO, 2023: CRD42023437808).

### Databases and search strategy

A literature search was conducted using Google Scholar, PubMed, and Hinari. All studies conducted on patterns of help-seeking behavior and associated factors among people with mental illness in Ethiopia were included. To identify relevant data on help-seeking behavior and associated factors among people with mental illness, comprehensive searches were conducted via the mentioned databases using the following search terms and phrases, which were combined using the “OR” and “AND” Boolean operators: “Pattern of help-seeking behavior’’ OR “Pattern of treatment-seeking behavior” OR “Health care-seeking behavior” OR “Help-seeking intention” OR “Help-seeking preferences” OR “perceived need” OR “Pathways to psychiatric care”, AND “Common mental disorders” OR “Mental illness” OR “Mental health problems” OR “Depression”, AND “Predictors” OR “Determinate factors” OR “Associated factors”, AND “Ethiopia”. Finally, all articles that were congruent with the topic of the review were retrieved and screened according to the inclusion criteria. The literature search was conducted from June 22, 2023 to December 20, 2023 and was performed by two independent researchers, with discrepancies resolved by discussion and consensus.

### Eligibility criteria

All available primary studies were searched. Initially, all observational studies that measured help-seeking behavior and its predictors among people with mental illness in Ethiopia and that were written in the English language were included. We did not place a limitation on the earliest time of publication. Qualitative studies, case series, and case reports were excluded.

### Study selection

All the articles retrieved were exported to EndNote version 7 reference manager, and duplicates were removed using the “find duplicates” function. Subsequently, screening and selection of the studies was conducted in two phases: first, the abstract and title were screened, and then the full text was reviewed. Based on screening of the title and abstract by two independent researchers, articles that reported on patterns of help-seeking behavior among people with mental illness were selected for full-text review. Any article deemed potentially eligible by either reviewer then underwent full-text review and was individually screened by both reviewers. In cases in which the researchers were unable to come to an agreement, a third researcher reviewed the article and resolved the disagreement.

### Data extraction

Data were extracted using a standardized data abstraction form, developed in an Excel spreadsheet. For each study, the following data were extracted: author’s name, publication year, study region, study setting, study design, sample size, response rate, and pattern of help-seeking behavior. For each study, data extraction was carried out by two researchers (SA and GW), and the extracted data were then cross-checked for inconsistencies. Disagreements were resolved with the involvement of a third reviewer.

### Primary outcome measure of interest

The primary outcome measure of interest was a positive pattern of help-seeking behavior with respect to formal modern treatment among people with mental illness, and the predictors of this behavior, in Ethiopia.

### Data analysis

All statistical analyses was performed using STATA version 17. A weighted inverse-variance random-effects model (54) was used to calculate the overall pooled pattern of positive help-seeking behavior with respect to modern treatment among people with mental illness and the predictors of such behavior. The presence of publication bias was tested for by examining the symmetry of a funnel plot, and Egger’s test (with a p-value threshold of <0.05) was also employed to determine whether significant publication bias was present (55). The percentage of total variation across studies due to heterogeneity was assessed based on the I^2^ statistic (56). Values of I^2^ = 25, 50, and 75% were taken to represent low, moderate, and high heterogeneity, respectively (56).

A p-value of <0.05 for the I^2^ statistic was taken to indicate significant heterogeneity (57, 58). To determine the influence of a single study on the overall meta-analysis, a sensitivity analysis was performed. Forest plots were created to estimate the effects of independent factors on the outcome variable, and a measure of association is also reported with the 95% CI. The odds ratio (OR) was the most frequently reported measure of association in the eligible primary studies.

To estimate the pooled OR effect, either a fixed effects or a random effects model can be used. A fixed effects model is appropriate when the results only apply to the studies included in the meta-analysis; there is one common fixed parameter and all studies estimate the same common fixed parameters; it is reasonable to consider the studies similar enough that there is a common effect; all the included studies used comparable methodology; and all samples were drawn from identical populations. In contrast, a random effects model is appropriate when the results extend beyond the included studies; there is no common, fixed parameter, and studies estimate different parameters; the studies are different and it is not reasonable to consider them to be examining a common effect; the included studies used different methodologies; or the samples were drawn from different populations. In the case of our review, the primary studies included used different methodologies and the samples were drawn from several independent populations, resulting in significant heterogeneity and variation in the true effect size across studies. Thus, a random effects model was used in this review.

## Results

### Search outcomes

Our exhaustive survey of both published and unpublished sources in the database search identified a total of 912 articles. All 912 articles were obtained via the database search. Among these, 585 articles were identified using Google Scholar, 291 using PubMed, and 36 using Hinari. A total of 729 duplicate articles were excluded. After screening of the remaining 183 articles based on their title and abstract, 83 articles were excluded due to not covering the topic of interest.

**Figure 1:**
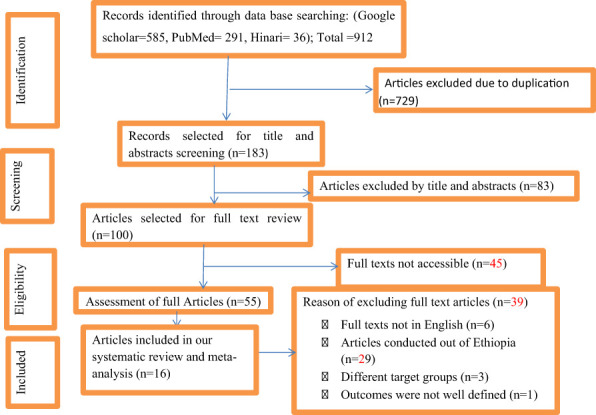
PRISMA flow diagram showing the results of the literature search.

Next, the remaining 100 articles were assessed for availability of the full text; full text content was available for only 55 of these. After full text review of these 55 articles, 39 were excluded due to a) not being written in English, b) reporting on a study conducted outside Ethiopia, c) reporting on a study with a different target group, or d) the outcomes not being well defined. Finally, articles reporting on a total of 16 empirical studies (4, 38–52) were included (**Figure 1**).

### Characteristics of the studies included

In this systematic review, a total of 7092 adults living with mental illness were included across all included studies, with approximately half (50.7%) being men. The publication year of the included studies ranged from 1999 to 2021. The smallest and largest sample sizes among the included studies were from studies conducted in the Southern Nations, Nationalities, and Peoples' Region (SNNPR); sample sizes ranged from 100 (47) to 1135 (49). All were cross-sectional studies (n = 16, 100%). The majority (n=12, 75%) of the included studies were community-based, while the remainder were conducted in an institutional setting. The response rates of the individual studies ranged from 90.1% to 100%, and the rates of help-seeking behavior among people with mental illness ranged from 16.2% (40) to 81.5% (44).

Regarding geographical region, five studies (4, 38, 40, 43, 45) were conducted in Oromia, five (41, 44, 48, 50, 52) in Amhara, five (39, 42, 46, 47, 49) in the SNNPR, and one (51) in the Tigray region (**Table 1**).

**Table 1:** Characteristics of the studies included.

S No	Author [Year]	Sex ratio		Region	Study setting	Study design	Sample size	Response rate	Prevalence
		F	M						
1	Belete A et al. [2019] (38)	101	94	Oromia	Institutional	CS	195	96%	33.3
2	Fekadu A et al. [2008] (39)	184	168	SNNPR	Community-based	CS	352	100%	35.3
3	Gebreegziabher Y et al. [2019] (40)	295	149	Oromia	Institutional	CS	444	90.1%	16.2
4	Hailemariam S et al. [2012] (48)	242	208	Amhara	Community-based	CS	450	98.7%	22.9
5	Kerebih H et al. [2017] (4)	163	82	Oromia	Community-based	CS	245	97.8%	49.4
6	Menberu M et al. [2018] (41)	129	97	Amhara	Community-based	CS	226	100%	25.66
7	Rathod SD et al. [2016] (42)	61	58	SNNPR	Community-based	CS	119	98.5%	23.7
8	Tesfaye Y et al. [2020] (43)	230	190	Oromia	Community-based	CS	420	99.3%	25.2
9	Yeshanew B et al. [2019] (44)	393	554	Amhara	Community-based	CS	947	98.23%	81.5
10	Alem A et al. [1999] (47)	12	88	SNNPR	Community-based	CS	100	100%	41
11	Girma E et al. [2011] (45)	146	238	Oromia	Community-based	CS	384	100%	35.2
12	Azale T et al. [2016] (46)	385	0	SNNPR	Community-based	CS	385	100%	49.9
13	Shumet S et al. [2021] (50)	343	489	Amhara	Community-based	CS	832	98.3%	78.7%
14	Negash A et al. [2020] (49)	448	687	SNNPR	Institutional	CS	1135	86.34%	70.5%
15	Teshager S et al. [2020] (51)	197	226	Tigray	Institutional	CS	423	100%	22.5%
16	Getaneh E et al. [2021] (52)	166	269	Amhara	Community-based	CS	435	97.2%	63.8%

### Thematic findings

Sixteen primary studies conducted in four regions of Ethiopia (Amhara, Oromia, the SNNPR, and Tigray) were included in this review.

In a community-based cross-sectional study conducted in Butajira in the SNNPR involving 100 individuals living with mental illness, it was found that 41% of them sought help from a modern health institution for their problems. Another community-based cross-sectional survey conducted in southern Ethiopia, in which 385 mothers with postpartum depression were included, revealed that nearly half (49.9%) of the respondents exhibited a positive pattern of help-seeking behavior with respect to modern treatment. In an institution-based cross-sectional study conducted among 195 patients with depression in Jima in the Oromia region, it was reported that only 33.3% of the participants sought help from modern health facilities. Factors significantly associated with positive help-seeking behavior were being employed (AOR = 4.24, 95% CI: 1. 31, 13.78), having a secondary education or above (AOR= 7.6, 95% CI: 2. 13, 27.11), having a family history of mental illness (AOR= 7.33, 95% CI: 2. 72, 19.80), having ideas about hurting oneself, awareness of the availability of psychiatric services at the hospital, and having previously engaged in positive help-seeking behavior. Similarly, a community based cross-sectional study conducted in the SNNPR comprising 352 patients with depression indicated that 35.3% of them exhibited a positive pattern of help-seeking behavior with respect to modern treatment. The odds of engaging in a positive pattern of help-seeking behavior with respect to modern treatment were increased by 6.27 times among respondents who were aware of the availability of psychiatry services compared with their counterparts (AOR= 6.27, 95% CI: 2. 05, 19.2).

A study conducted in the Oromia region, in which 384 participants living with major depressive disorder, schizophrenia, and other psychotic disorders were included, indicated that 35.2% of them sought help from modern health services. Similarly, a study carried out in Jima in the Oromia region reported that, among 444 respondents with common mental disorders, only 16.2% of them sought help with their problems from a modern health facility. This study indicated that respondents who had a very poor overall level of satisfaction with life were less likely to seek help (p = 0.011) compared with those who had a very good level of satisfaction with life. This study also found that having no previous history of help-seeking behavior was significantly associated with seeking help for their problems (i.e., CMDs; p<0.001). Furthermore, a study of 450 depressed people found that nearly one-quarter (22.9%) of the respondents sought care from modern health institutions for their problems. The findings of this study also revealed that respondents who believed mental illnesses require treatment (AOR= 2.33, 95% CI: 1.25, 4.35), had a secondary education or above (AOR= 2.6, 95% CI: 1.23, 5.43), had engaged in suicidal behavior (AOR= 1.82, 95% CI: 1.08, 3.04), were aware of the availability of modern treatment services (AOR= 1.98, 95% CI: 1.18, 3.31), or had a history of positive help-seeking behavior were more likely to seek help from a health care institution for their problems.

Additionally, in the community-based cross-sectional study conducted in the southern Oromia region among 245 individuals with common mental disorders, it was reported that half (49.9%) of the patients sought professional help for their illness. Factors significantly associated with increased help-seeking behavior for these disorders were: being 48 years old or older (AOR= 10.18, 95% CI: 1.45,71.49); being female (AOR=6.23, 95% CI: 2.41, 16.12); not using khat (AOR= 3.12, 95% CI: 1.28, 7.51), alcohol (AOR= 5.02, 95% CI: 1.55, 16.20), or cigarettes (AOR= 5.24, 95% CI; 1.45, 18.95); and having chronic physical illness (AOR= 10.23, 95% CI: 3.68, 28.48). A cross-sectional community-based study conducted among 226 patients with depression indicated that only 25.66% of the patients sought help from health professionals for their condition. The study also found that being female (AOR = 2.77, 95% CI: 1.280, 5.99), current alcohol consumption (AOR = 2.74, 95% CI: 1.27, 5.94), co-morbid medical–surgical illness (AOR = 4.49, 95% CI: 1.823, 11.07), and perceiving depression as an illness (AOR = 2.44, 95% CI: 1.26, 4.93) were all significantly associated with help-seeking behavior among the participants.

A population-based cross-sectional survey of 119 depressed individuals conducted in the SNNPR indicated that 23.7% of these individuals sought help from health professionals. Respondents who believed that mental illness required treatment (AOR=2.4, 95% CI: 1.26, 4.67), had completed a secondary education or above (AOR=8.7, 95% CI: 5.08, 15.11), or had a family history of mental illness (AOR=7.5, 95% CI: 2.69, 8.76) were more likely to seek help from health professionals. It was also observed that a survey involving 420 people living with mental illnesses in the Jima, Oromia region found that the majority (74.8%) of respondents favored non-medical approaches to treatment. In comparison to other findings, respondents in the Amhara region exhibited a higher rate of positive help-seeking behavior with respect to modern treatment, at 81.5% (44), 78.5% (50), and 63.8% (52). A relatively high rate of help-seeking behavior with respect to modern treatment was also documented in another study conducted in the SNNRP, at 70.5% (49). Factors with a positive impact on help-seeking intent were: having the belief that mental illness requires treatment (AOR = 3.42, 95% CI: 1.1, 10.55), being 25–34 years old (AOR = 1.46, 95% CI: 1.02, 2.09), having strong social support (AOR = 1.85, 95% CI: 1.25, 2.72), having previously engaged in positive help-seeking behavior (AOR=1.93, 95% CI: 1.07, 3.47), having comorbid medical illness (AOR=2.11, 95% CI: 1.22, 3.66), and a higher perceived severity of mental illness (AOR=2.7, 95% CI: 1.15, 6.34) (44). Finally, another study conducted in the Tigray region, which included 423 individuals living with mental illnesses, found that approximately one-quarter of them (22.5%) exhibited positive help-seeking behavior with respect to modern treatment.

### Operational definition of variables 

In this review, a pattern of positive help-seeking behavior was regarded as a preference on the part of the patient to be treated by modern medical services, as opposed to other forms of informal treatment, such as traditional religious treatment.

### Quality appraisal of the included studies

Two independent reviewers (SA and TM) appraised the quality of the studies included and scored them on the validity of their results. The quality of each study was evaluated using the Joanna Briggs Institute (JBI) quality appraisal criteria (59). All sixteen studies included (4, 38–52) were appraised as cross-sectional studies using JBI checklist. Among the sixteen studies, eleven studies scored “yes” on seven of eight questions (87.5%; low risk), three studies scored “yes” on six of eight questions (75%; low risk), and the remaining two studies scored “yes” on five of eight questions (62.5%; low risk) (**Supplementary Table 2**). Studies were considered to be at low risk if they scored 50% or higher on the quality assessment indicator. After conducting a thorough quality appraisal, we determined that the primary studies included in our analysis displayed a high level of reliability in their methodological quality scores. All the studies included in this review scored between 5 and 7 out of a total of 8 points. Thus, all studies included in the review (4, 38–52) were of high quality.

### Risk of bias assessment

The adopted assessment tool (60) was used to assess the risk of bias. This tool consists of ten items that assess four areas of bias, including internal validity and external validity. Items 1–4 evaluate selection bias, non-response bias, and external validity; items 5–10 assess measure bias, analysis-related bias, and internal validity. Among all sixteen included studies, five studies scored “yes” on nine of the ten questions, and the remaining eleven studies scored “yes” on eight of the ten questions. A study was categorized as “low risk” when eight or more questions had a “yes” response, “moderate risk” when six or seven questions had a “yes” response, and “high risk” when five or fewer questions had a “yes” response. Therefore, all studies included (4, 38–52) had low risk of bias (i.e., were of high quality) (**Supplementary Table 3**).

### Meta-analysis

#### Pooled data on pattern of positive help-seeking behavior

**Figure 2:**
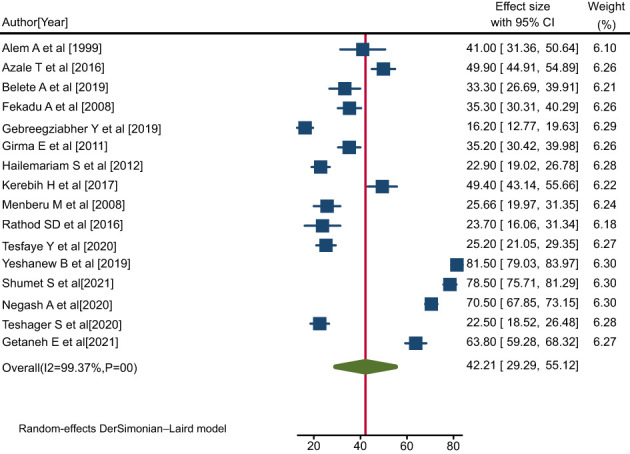
Forest plot showing the pooled rate of positive help-seeking behavior with respect to formal modern treatment among people with mental illness in Ethiopia, 2023.

Overall, sixteen eligible primary studies (4, 38–52) were included in the final meta-analysis. Data on rates of help-seeking behavior among people with mental illness were obtained from all sixteen primary studies included (4, 38–52), while data regarding the predictors of patterns of positive help-seeking behavior were also obtained from ten of these studies. The pooled rate of positive help-seeking behavior among people with mental illness was 42.21% (95% CI: 29.29, 55.12; I^2^ = 99.37%, P=0.00) (**Figure 2**).

#### Publication bias

**Figure 3:**
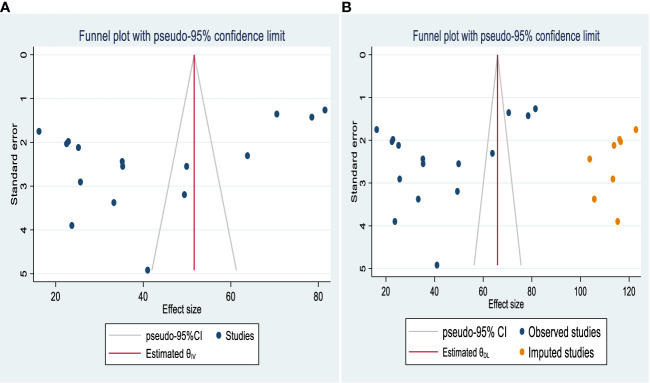
Funnel plot showing publication bias in studies of help-seeking behavior among people with mental illness in Ethiopia **(A)** before and **(B)** after adjustment using trim-and-fill analysis.

Publication bias was examined via both funnel plots and Egger’s regression test. Funnel plots exhibited an asymmetric shape, which indicates the presence of publication bias among the studies included (**Figure 3A**), and the result of Egger’s regression test was a value of 0.167. Duval and Tweedie nonparametric trim-and-fill analyses were conducted to correct publication bias among the studies. This indicated that publication bias would be controlled if eight additional studies were included in the review (**Figure 3B**).

### Investigation of heterogeneity

The I^2^ statistic corresponding to the forest plot indicated marked heterogeneity among the included studies (I^2^ = 99.37%, P=0.00) (**Figure 2**). Hence, sensitivity and sub-group analyses were carried out to minimize heterogeneity.

### Sensitivity analysis

**Figure 4:**
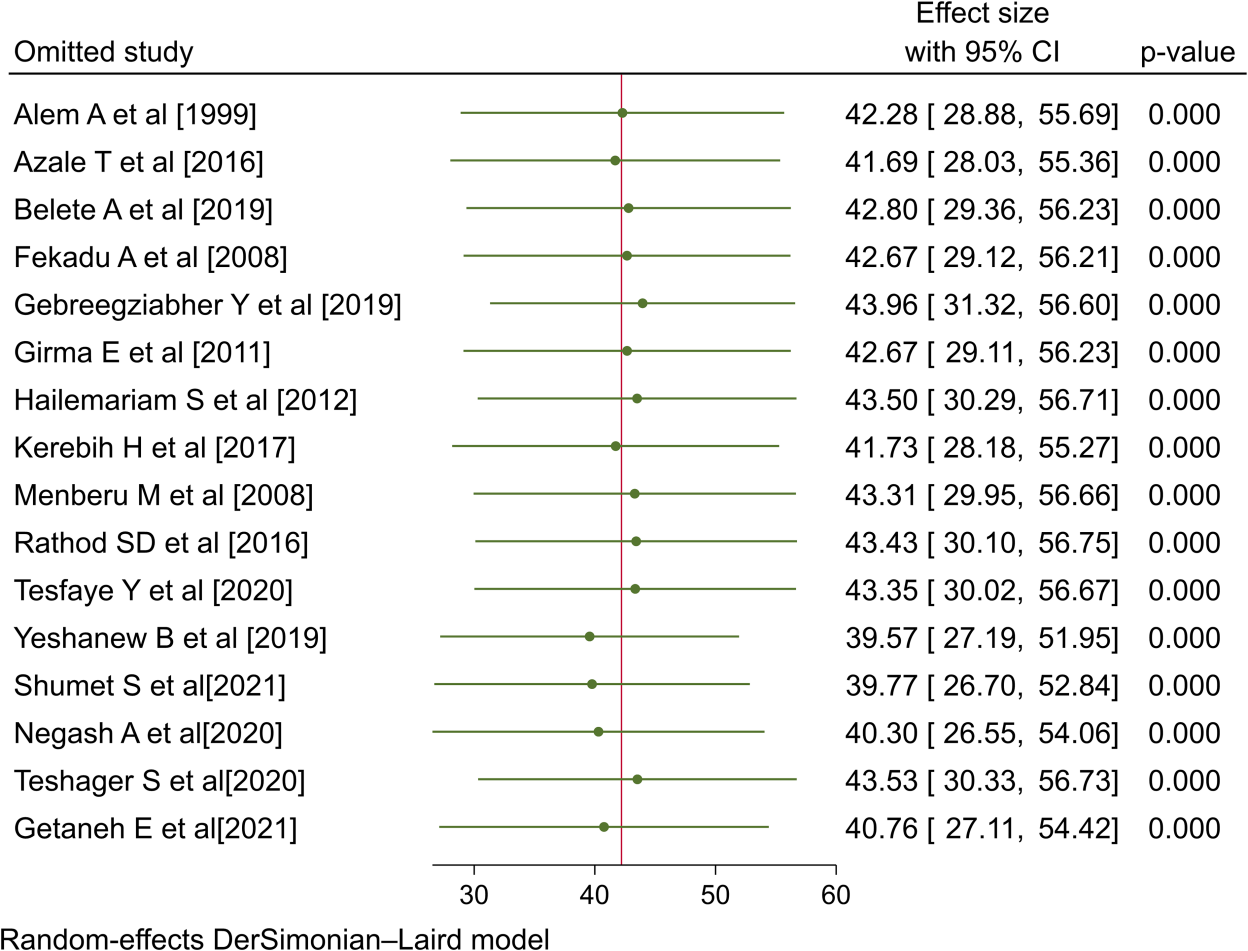
Sensitivity analysis for the rate of positive help-seeking behavior with respect to formal modern treatment among people with mental illness in Ethiopia, 2023.

In order to determine the influence of each specific study on the overall meta-analysis, we conducted a sensitivity analysis. The forest plot showed that the estimated rate with a single study omitted was close to the combined estimate in each case, which implies that no single study had a major effect on the overall pooled estimate. Thus, it was demonstrated that no single study had a significant impact on the overall outcome of the meta-analysis (**Figure 4**).

### Subgroup analyses

As part of this meta-analysis, we conducted subgroup analyses based on study region, sample size (<400 vs. >=400), study setting (institutional vs. community-based), and publication year (before 2019 vs. 2019 or later).

#### Subgroup analysis by region

**Figure 5:**
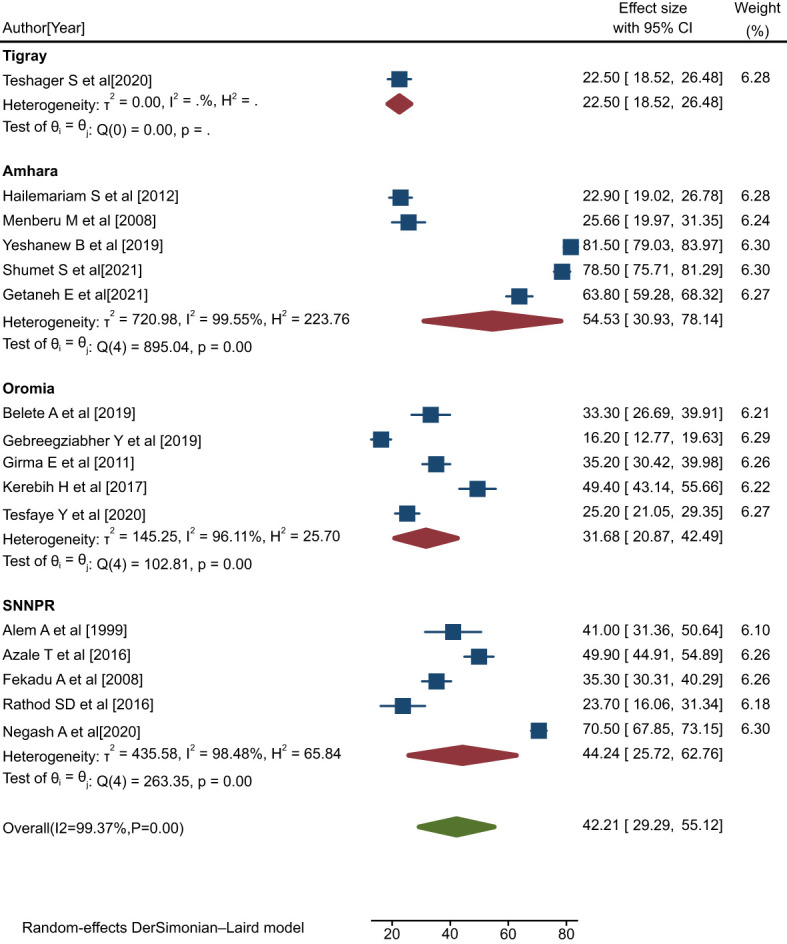
Forest plot showing subgroup analysis by region for rates of help-seeking behavior among people with mental illness in Ethiopia, 2023.

In the sub-group analysis by region, it was found that people with mental illness exhibited a relatively high pooled rate of positive help-seeking behavior in the Amhara region (54.53%; 95% CI: 30.93, 78.14; I^2^ = 99.55%, P=0.00) compared with the SNNR (44.24%; 95% CI: 25.72, 62.76; I^2^ = 98.48%, P=0.00), the Oromia region (31.68%; 95% CI: 20.87, 42.49; I^2^ = 96.11%, P<0.00), and the Tigray region (25.5%; 95% CI: 18.52, 26.48; I^2^ = 0) (**Figure 5**).

#### Subgroup analysis by study setting

**Figure 6:**
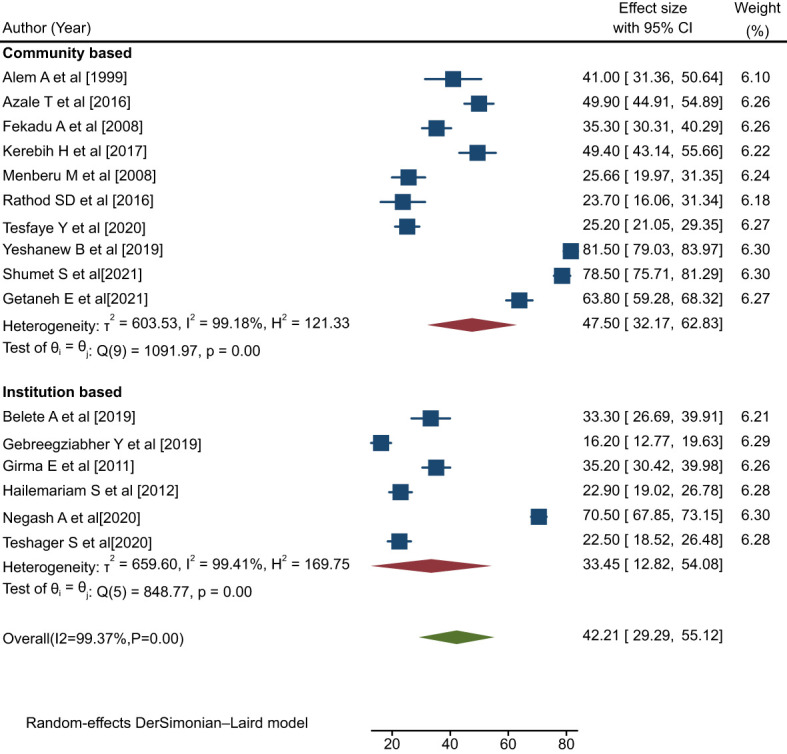
Forest plot showing the subgroup analysis by study setting for rates of help-seeking behavior among people with mental illness in Ethiopia, 2023.

The findings indicated that studies conducted in a community-based setting found relatively high rates of help-seeking behavior with respect to modern treatment (47.50%; 95% CI: 32.17, 62.83; I**^2^ =** 99.18%, p=0.00) compared with those conducted in an institutional setting (33.45%; 95% CI: 12.82, 54.08: I**^2^ =** 99.41%, p=0.00) (**Figure 6**).

#### Subgroup analysis by sample size

**Figure 7:**
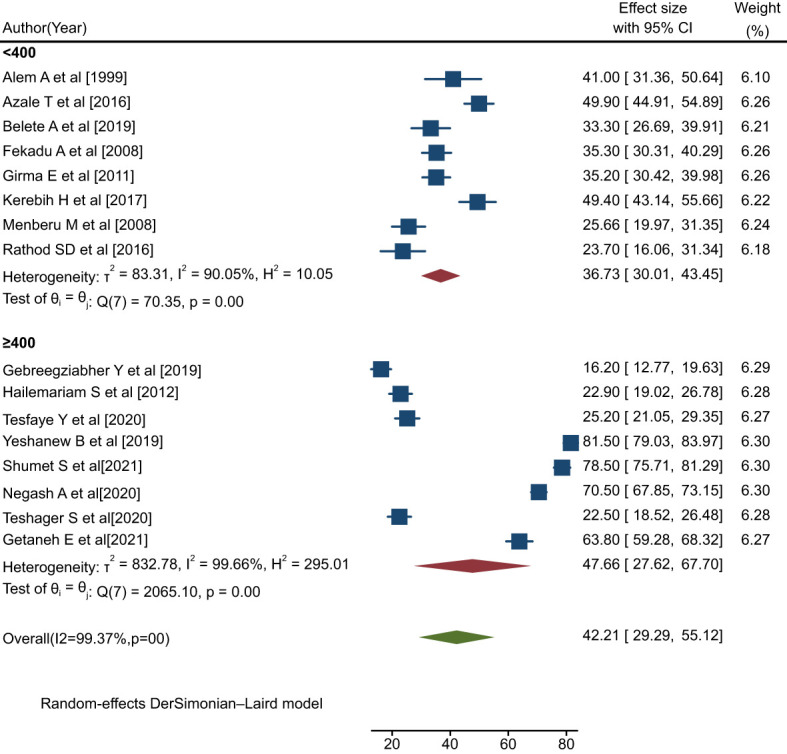
Forest plot showing the subgroup analysis by sample size for rates of help-seeking behavior among people with mental illness in Ethiopia, 2023.

The findings of this analysis indicated that studies with a sample size of <400 observed relatively low rates of help-seeking behavior (36.73%; 95% CI: 30.01, 43.45; I^2^ = 90.05%, p=0.00) compared to those with a sample size of >=400 (47.66%; 95% CI: 27.62, 67.70; I^2^ = 99.66%, p=0.00) (**Figure 7**).

#### Subgroup analysis by publication year

**Figure 8:**
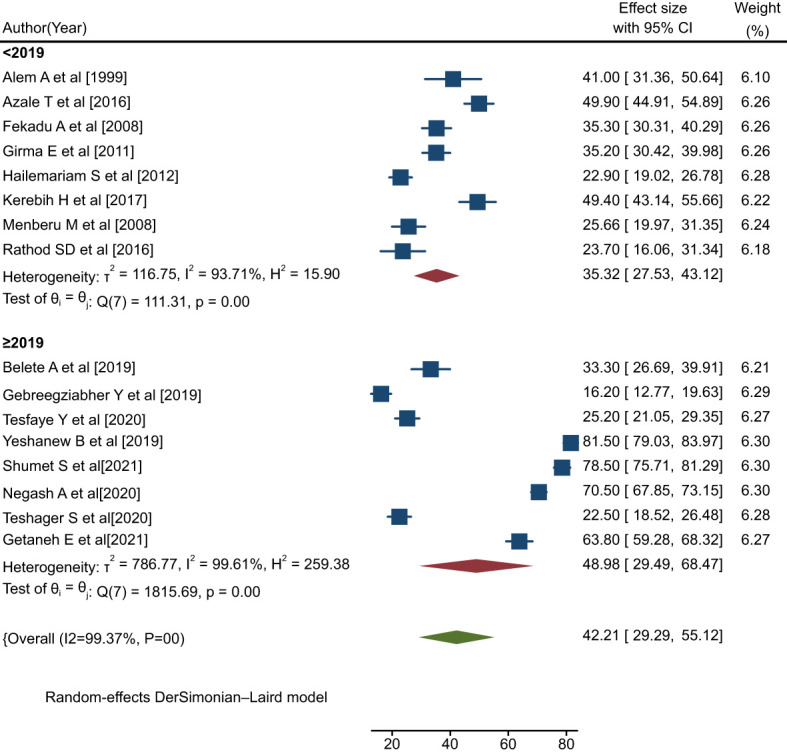
Forest plot showing the subgroup analysis by publication year for rates of help-seeking behavior with respect to formal modern treatment among people with mental illness in Ethiopia, 2023.

The pooled rate of positive help-seeking behavior among people with mental illness in studies conducted before the year 2019 was 35.32% (95% CI: 27.53, 43.12; I^2^ = 93.71%, P=0.00), which was lower than the rate observed in studies conducted in 2019 or later (48.98%; 95% CI: 29.49, 68.47; I^2^ = 99.61%, P=0.00) (**Figure 8**).

These subgroup analyses revealed that the heterogeneity among the primary studies may be attributable to differences in region, sample size, study setting, and publication year.

### Factors associated with positive help-seeking behavior

**Figure 9:**
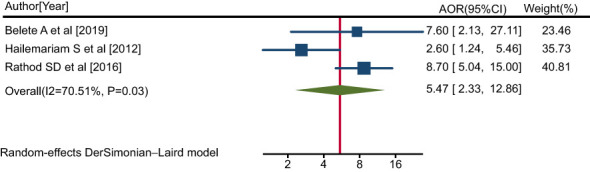
Forest plot showing the adjusted odds ratios obtained in different studies, with corresponding 95% CIs, for the association between having a secondary education or above and rates of positive help-seeking behavior with respect to medical treatment among people with mental illness. The midpoint and length of each segment indicate the AOR and 95% CI, respectively; the diamond shape represents the combined AOR over all studies.

In this review, three studies (38, 42, 48) reported a significant association between having a secondary education or above and a pattern of positive help-seeking behavior with respect to medical treatment among people with mental illness. The pooled AOR for having attained a secondary education or above for the rate of positive help-seeking behavior with respect to medical treatment among people with mental illness was 5.47 (95% CI: 2.33, 12.86; I^2^ = 70.51%; P=0.03) (**Figure 9**).

**Figure 10:**
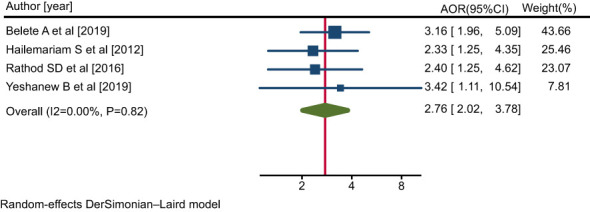
Forest plot showing the adjusted odds ratios obtained in different studies, with corresponding 95% CIs, for the association between the belief that mental illness requires treatment and rates of positive help-seeking behavior with respect to medical treatment among people with mental illness. The midpoint and length of each segment indicate the AOR and 95% CI, respectively; the diamond shape represents the combined AOR over all studies.

Four studies (38, 42, 44, 48) indicated that a belief that mental illness requires treatment was significantly associated with a pattern of positive help-seeking behavior with respect to medical treatment among people with mental illness. The pooled AOR for the belief that mental illness requires medical treatment for the rate of positive help-seeking behavior among people with mental illness was 2.76 (95% CI: 2.02, 3.78; I^2^ = 0.00%; P=0.82) (**Figure 10**).

**Figure 11:**
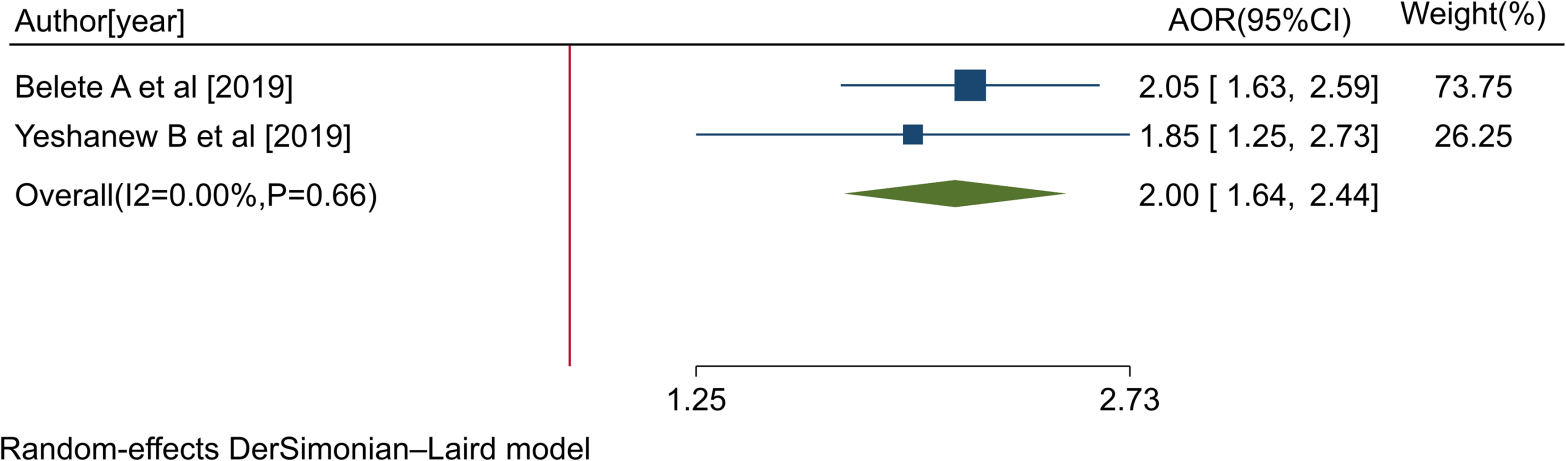
Forest plot showing the adjusted odds ratios obtained in different studies, with corresponding 95% CIs, for the association between strong social support and rates of positive help-seeking behavior with respect to medical treatment among people with mental illness. The midpoint and the length of each segment indicate the AOR and 95% CI, respectively; the diamond shape represents the combined AOR over all studies.

Two studies (38, 44) indicated a significant association between strong social support and a pattern of positive help-seeking behavior with respect to medical treatment among people with mental illness. The pooled AOR for strong social support for the rate of positive help-seeking behavior with respect to medical treatment among people with mental illness was 2.00 (95% CI: 1.64, 2.44; I2 = 0.00%; P=0.66) (**Figure 11**).

Two studies (38, 42) revealed a significant association between family history of mental illness and pattern of good help seeking behavior of people with mental illness to medical treatment. The pooled AOR of pattern of good help seeking behavior of people with mental illness to medical treatment and who had family history of mental illness was 2.68 (95%CI: 1.38, 3.97,I^2^ = 74.44%, P<0,048).

Three studies (39, 40, 48) reported a significant association between people living with mental illness who know the availability of medical treatment and their pattern of good help seeking behavior to medical treatment. The pooled AOR pattern of good help seeking behavior of people with mental illness to medical treatment and who know the availability of medical treatment was 2.92(95%CI: 1.56, 5.46,I^2^ = 44.83,p<0.163).

Four studies (38, 40, 44, 48) showed a significant association between people who had previous good help seeking behavior and pattern of good help seeking behavior to the medical treatment. The pooled AOR of pattern of good help seeking behavior to the medical treatment and who had previous good help seeking behavior was 3.28 ((95%CI: 1.63, 6.60).

Three studies (4, 41, 44) also showed significant associations between people have comorbid disorder and pattern of good help seeking behavior to the medical treatment. The pooled AOR of pattern of good help seeking behavior to the medical treatment of patients with mental illness and who had comorbid disorder was 4.25 (95%CI: 1.69, 10.66).

Two studies (4, 41) reported a significant association between individuals who did not use alcohol and their pattern of good help seeking behavior to the medical treatment. The pooled AOR of pattern of good help seeking behavior to the medical treatment and individuals who did not use alcohol was 3.29 (95%CI: 1.73, 6.27).

Two studies (41, 44) reported a significant association between individuals who had perceived severity of mental illness and their pattern of good help seeking behavior to the medical treatment. The pooled AOR of pattern of good help seeking behavior to the medical treatment and individuals who had perceived severity of mental illness was 2.54 (95%CI: 1.490, 4.33).

Thus, people with mental illness with a secondary school education or above were 5.47 times more likely to exhibit a positive pattern of help-seeking behavior with respect to modern treatment compared with participants who were illiterate.

People with mental illness who believed that mental illness requires treatment were 2.76 times more likely to exhibit a positive pattern of help-seeking behavior with respect to modern treatment compared with participants who did not believe this.

Individuals living with mental illness who had strong social support were 2.00 times more likely to exhibit a positive pattern of help-seeking behavior with respect to modern treatment compared with respondents who had poor social support.

People with mental illness who had a family history of mental illness were 2.68 more likely to seek help from modern health facilities compared with those who had no family history of mental illness.

Likewise, people with mental illness who had knowledge of the availability of modern treatment for mental illness were 2.92 times more likely to exhibit a positive pattern of help-seeking behavior with respect to modern treatment compared with those who were unaware of it.

Similarly, people with mental illness who had previously engaged in positive help-seeking behavior with respect to modern treatment were 3.28 times more likely to exhibit a positive pattern of help-seeking behavior with respect to modern treatment compared with those who had previously engaged in negative help-seeking behavior.

The likelihood of exhibiting a positive pattern of help-seeking behavior was 4.25 times greater among respondents who had comorbid disorders compared with those who did not have such disorders.

Additionally, individuals with mental illness who did not use alcohol were 3.29 times more likely to exhibit a positive pattern of help-seeking behavior with respect to modern treatment compared with those who did use alcohol.

Finally, people with mental illness who perceived their mental illness to be severe were 2.54 times more likely to exhibit a positive pattern of help-seeking behavior with respect to modern treatment compared with respondents who did not have this perception.

## Discussion

In a society where health care facilities are scarce and overburdened with people suffering from communicable diseases, traditional healers will continue to provide therapy for mental disorders for some time. In Ethiopia, traditional methods have been preferred over modern medicine for treating neuropsychiatric disorders, in contrast to the preference to treat physical problems with modern medicine. As a result, working closely with traditional healers would provide primary health workers with a better chance of gaining community acceptability and changing certain detrimental traditional practices.

Thus, we conducted the first systematic review and meta-analysis on patterns of help-seeking behavior with respect to formal modern treatment among people with mental illness, and the predictors of this behavior, in Ethiopia.

We identified a total of 912 articles, among which, 16 studies provided data suitable to be included in the systematic review and meta-analysis. In this review, a total of 7092 adults suffering from mental illness were included across the articles included. The studies included in this analysis were published between 1999 and 2021, with sample sizes ranging from 100 to 1135. All were cross-sectional studies (n = 16; 100%). The majority of the studies included (n=12, 75%) were community-based, with the remainder being based in institutional settings. Individual studies had response rates ranging from 90.1% to 100%, and the rates of help-seeking behavior among people with mental illness ranged from 16.2% to 81.5%. According to individual studies, individuals in the Amhara region exhibited relatively high rates of positive help-seeking behavior, whereas those in the Oromia region had the poorest rates of help-seeking behavior with respect to modern treatment.

The results of our meta-analysis indicated that the overall pooled rate of positive help-seeking behavior with respect to modern treatment among people with mental illness was 42.21% (95% CI: 29.29, 55.12); I^2^ = 99.37%; p=0.00), which was in line with the findings of research conducted in Singapore (32%) (21), Rwanda 36% (17), Indonesia 46.2% (22), China (52%) (23), and Brazil 51.1% (24). However, the findings of this study indicated a lower rate compared to studies conducted in the Netherlands (65%) (25), Zanzibar (58.9%) (26), Hunan, China (64.6%) (27), Australia(85%) (28), and Egypt (90.3%) (29). In contrast, the rate indicated in the current study was higher than that observed in research conducted in other China-based studies (12.7%, 28.2%) (19, 30), in Somerset, England (28%) (18), and in another Brazilian study (28.4%) (20). This variation might be due to methodological, sample size, and cultural differences.

Furthermore, the finds of this study revealed that individuals with mental illness who had attained education at the secondary school level or above exhibited a positive pattern of help-seeking behavior with respect to modern treatment. This is consistent with studies conducted in Canada and China (19, 61, 62). This may be because people with higher educational attainment have a better understanding of mental health and more favorable attitudes toward those who suffer from mental illness. Consequently, instead of staying at home and relying on informal forms of treatment, they seek assistance from medical facilities, medical specialists, and psychiatrists. Participants who had comorbid medical illness were also more likely to seek help from modern facilities for their mental health issues compared with their counterparts, which is supported by previous research conducted in Brazil, Canada, and China (20, 23, 63). The likelihood of readmission to psychiatric hospital is higher among individuals with mental illnesses who also have medical comorbidities than in individuals without such conditions. This has an impact on the patient’s finances, their overall health, and the health of their family, which makes them more likely to seek out formal modern treatment for their problems.

Similarly, people with mental illness who believed that mental illness requires treatment showed a positive pattern of help-seeking behavior with respect to modern treatment compared with those participants who did not believe that mental illness requires treatment. It is true that individuals who feel that mental illness requires medical attention are more likely to seek help for their mental health issues at a modern health care facility.

People with mental illness who had strong social support networks were more likely to seek treatment for their mental health issues from a modern health care institution. This has also been reported in research conducted in China (30). It has been demonstrated that family members or friends are crucial in supporting patients as they cope with their disease. They can also assist by suggesting that individuals with mental illnesses finally seek out professional assistance. In addition, this may be because individuals with social support networks are more likely to receive financial support and to obtain more accurate information about the availability of formal medical treatments, both of which encourage patients to seek out medical professionals for assistance. Furthermore, individuals who have a history of mental illness may need to be treated at a modern health care facility for their subsequent mental ill health issues due to their fear of relapsing into a serious condition.

Additionally, this review showed that respondents who were aware that modern formal treatments were available for mental illness were more likely to seek help from mental health professionals for their issues, in contrast to those who were unaware of these treatments. This has also been demonstrated in China, Uganda, and Zanzibar, and in a study conducted in the UK and Nigeria (26, 30, 64). Those with a history of mental illness also exhibited a positive pattern of help-seeking behavior with respect to modern treatment. A history of mental health issues makes people more inclined to seek assistance because they are afraid of suffering, functional impairment, and other issues that may arise from being admitted to the hospital.

Furthermore, those who had a history of positive help-seeking behavior with respect to modern therapy for their condition were more likely to have sought care from a modern health institution for a later episode. This finding was in agreement with previous research conducted in China, Western Australia, and Switzerland (65–67). One explanation for this could be that individuals with mental illnesses who have a history of a positive pattern of help-seeking behavior with respect to current treatments are more likely to improve and may also be satisfied with their prior interactions. This makes it possible for them to make use of the service during their next episode.

Similarly, those with mental illness who had a family history of mental illness exhibited a more positive pattern of help-seeking behavior with respect to modern treatment than those without such a history. This finding is supported by those of studies conducted in Egypt and Hunan, China (27, 29). This correlation may be explained by the possibility that individuals with a family history of mental illnesses are more aware of the resources for mental health care that are available and are less likely to feel stigma in relation to obtaining treatment.

In this study, we found that respondents who consumed alcohol had poor rates of engaging in help-seeking behavior with respect to formal modern treatment. This finding is supported by previous work (68). Since alcohol is a depressive, it slows down neuronal activity and brain function. Like other depressants, it hinders and reduces mental and physical activities. This means that it can disrupt the balance of neurotransmitters in the brain, which can affect the patient’s mood, thoughts, and behavior, and slows down how they process information. Broadly speaking, alcohol consumption has an impact on the patient’s drive to engage in meaningful actions in life, such as seeking treatment for mental health issues at a modern health care institution.

When comparing respondents who perceived their mental illness as serious to those who did not, we found that the former group had a higher likelihood of exhibiting a positive pattern of help-seeking behavior with respect to modern health services. This finding was in line with those of the study conducted in Zanzibar (26). It is true that people’s perceptions have an impact on their general behavior. In this case, those who view mental illness as a severe condition are more likely to exhibit positive patterns of help-seeking behavior with respect to modern treatment for their illness.

### Strengths and limitations of the study

This review has a number of positive features. An extensive search was performed. Three authors participated in the quality assessment. This review is also the first of its kind to present the aggregated results of multiple studies conducted in Ethiopia, which strengths the body of knowledge on patterns of positive help-seeking behavior among people living with mental illness and the predictors of this behavior. This study represents a significant improvement over the primary studies in that large number of study participants (n = 7092) from across sixteen studies were included. Moreover, all the studies included in the review were of good quality.

Despite its strengths, this review has limitations that should be noted for the interpretation of the results. All the included studies analyzed were cross-sectional studies. This review included some studies with small sample sizes, which may have influenced the conclusions. Our search was confined to papers published in English, resulting in the exclusion of some important studies. Finally, since this review included research conducted in only four regions of Ethiopia (Amhara, Oromia, the SNNPR, and Tigray), it is difficult to generalize the findings to all Ethiopians living with mental illness.

## Conclusions

The overall pooled rate of positive help-seeking behavior with respect to formal modern treatment among people living with mental illness in Ethiopia was low. Having attained a secondary education or above, believing that mental illness requires treatment, having strong social support, having a family history of mental illness, having awareness of the availability of medical treatment, having previously exhibited positive help-seeking behavior, having comorbid disorders, not using alcohol, and perceiving one’s mental illness to be severe were significantly associated with a positive pattern of help-seeking behavior. The findings indicated that the majority of Ethiopian people living with mental illness either seek help via informal forms of treatment, such as visiting traditional healers or obtaining religious treatment, or decline to receive any kind of treatment and stay at home.

### Recommendations for clinical practice

Mobilization of the community should be encouraged via regular public awareness campaigns regarding mental illness and the availability of evidence-based and effective modern treatment in Ethiopia. Likewise, the design of effective community-based mental health interventions is recommended in order to improve public attitudes and rates of help-seeking behavior in relation to mental health problems. Furthermore, it is important to work with closely with providers of informal treatment for mental illness so as to increase awareness of mental illness, its severity, and the availability of evidence-based and effective modern treatment.

### Policy implications

The findings of this review have revealed that the majority of individuals suffering from mental illnesses prefer informal forms of treatment and only approach modern health care institutions when traditional treatment has failed repeatedly. As a result, the Ethiopian government should place a high priority on raising levels of knowledge of the causes of mental illness and the availability of evidence-based treatment options through expanded community-based mental health education. Furthermore, it is recommended that a mental health day be celebrated nationwide in order to increase community awareness and to instill a more favorable attitude toward modern therapy.

### Scope for further research

This research has highlighted numerous implications for future studies in Ethiopia regarding interventions aiming to boost help-seeking behavior and, as a result, to minimize the treatment gap for mental health disorders. Research in other regions of Ethiopia on help-seeking behaviors with respect to modern treatment is also recommended. Moreover, it would be beneficial to conduct qualitative studies with key community informants, including traditional healers, religious leaders, etc. This would help in exploring the barriers to access to formal mental health services experienced by people living with mental illness in the community.

## Author contributions

SA: Writing – original draft, Methodology, Funding acquisition, Formal analysis, Conceptualization, Writing – review & editing. KH: Writing – review & editing, Methodology, Formal analysis, Data curation. BK: Writing – review & editing, Resources, Validation, Formal analysis, Data curation. GW: Writing – review & editing, Resources, Validation, Formal analysis, Data curation. GB: Writing – review & editing, Visualization, Project administration, Validation, Supervision, Formal analysis, Data curation. DF: Writing – review & editing, Funding acquisition, Validation, Supervision, Formal analysis, Data curation. DE: Writing – review & editing, Formal analysis, Data curation. GL: Writing – original draft, Software, Formal analysis, Data curation. BM: Writing – review & editing, Resources, Formal analysis, Data curation. AT: Writing – review & editing, Project administration, Methodology, Data curation. KS: Writing – review & editing, Software, Project administration, Data curation. AB: Project administration, Methodology, Data curation, Writing – review & editing. ES: Writing – review & editing, Methodology, Investigation, Formal analysis, Data curation. TM: Software, Project administration, Writing – review & editing.

## Supplementary material

Supplementary Table 1 PRISMA checklist.

Supplementary Table 2 Quality and risk of bias assessments of the included studies.
