# Supplementary material for: Patterns of help-seeking behavior among people with mental illness in Ethiopia: a systematic review and meta-analysis
Source: Front Psychiatry. 2024 Mar 18;15:1361092. doi: 10.3389/fpsyt.2024.1361092 (PMC10982478; doi:10.3389/fpsyt.2024.1361092)
Supplement: Supplementary Table 2 — Quality and risk of bias assessments of the included studies. [file Table_2.docx]

**Supplemental Table 2: Quality assessment of the included studies using the Joanna Briggs Institute (JBI) quality appraisal criteria**

| 1. **For cross-sectional studies** | | | | | | | | | | | | | | | | |
| --- | --- | --- | --- | --- | --- | --- | --- | --- | --- | --- | --- | --- | --- | --- | --- | --- |
| **S/N** | **Author [Year]** | **Criteria** | | | | | | | | | | | **Scores** | | | **Overall quality** |
|  |  | Clearly defined inclusion criteria | Describing the study settings participants | Valid &reliable exposure measurement | Objective &standard criteria for measurement | Identified confounder | | Strategies to deal with confounder | Valid & reliable outcome measurement | | Appropriate statistical analysis |  | | |  | |
|  | Belete A et al [2019] | Y | Y | Y | Y | N | | N | Y | | Y | 6 | | | Low risk | |
|  | Fekadu A et al [2008] | Y | Y | Y | Y | N | | Y | Y | | Y | 7 | | | Low risk | |
|  | Gebreegziabher Y et al [2019] | Y | Y | Y | Y | N | | Y | Y | | Y | 7 | | | Low risk | |
|  | Hailemariam S et al [2012] | N | Y | Y | Y | N | | Y | N | | Y | 5 | | | Low risk | |
|  | Kerebih H et al [2017] | Y | Y | Y | Y | N | | Y | Y | | Y | 7 | | | Low risk | |
|  | Menberu M et al [2018] | N | Y | Y | Y | N | | Y | Y | | Y | 6 | | | Low risk | |
|  | Rathod SD et al [2016] | Y | Y | Y | Y | N | | Y | Y | | Y | 7 | | | Low risk | |
|  | Tesfaye Y et al [2020] | N | Y | Y | Y | N | | Y | Y | | N | 5 | | | Low risk | |
|  | Yeshanew B et al [2019] | Y | Y | Y | Y | N | | Y | Y | | Y | 7 | | | Low risk | |
|  | Alem A et al [1999] | Y | Y | Y | Y | N | | Y | Y | | Y | 7 | | | Low risk | |
|  | Girma E et al [2011] | Y | Y | Y | Y | N | | Y | Y | | Y | 7 | | | Low risk | |
|  | Azale T et al [2016] | Y | Y | Y | Y | N | | Y | Y | | Y | 7 | | | Low risk | |
|  | Shumet S et al[2021] | Y | Y | Y | N | N | | Y | Y | | Y | 6 | | | Low risk | |
|  | Negash A et a(2020] | Y | Y | Y | Y | N | | Y | Y | | Y | 7 | | | Low risk | |
|  | Teshager S et al[2020] | Y | Y | Y | Y | N | | Y | Y | | Y | 7 | | | Low risk | |
|  | Getaneh E et al[2021] 63.8 | Y | Y | Y | Y | N | | Y | Y | | Y | 7 | | | Low risk | |
| ***Percentage (%) of ʺYesʺ*** | | | | | | | *8/10=80%* | | | *7/10=70%* | | | | *8/10=80%* | | |

Note: Y, yes; N, No

**Supplemental Table 3: Risk of bias assessment of the included studies**

| **S/N** | **Author [Year]** | **Criteria** | | | | | | | | | | **Scores** | **Overall risk of bias** |
| --- | --- | --- | --- | --- | --- | --- | --- | --- | --- | --- | --- | --- | --- |
|  |  | **External validity** | | | | **Internal validity** | | | | | |  |  |
|  |  | **Q1** | **Q2** | **Q3** | **Q4** | **Q5** | **Q6** | **Q7** | **Q8** | **Q9** | **Q10** |  |  |
|  | Belete A et al [2019] | Y | Y | N | Y | Y | Y | Y | Y | Y | Y | 9 | Low risk |
|  | Fekadu A et al [2008] | Y | Y | Y | Y | Y | Y | Y | Y | N | Y | 9 | Low risk |
|  | Gebreegziabher Y et al [2019] | Y | Y | N | Y | N | Y | Y | Y | Y | Y | 8 | Low risk |
|  | Hailemariam S et al [2012] | Y | Y | N | Y | Y | Y | N | Y | Y | Y | 8 | Low risk |
|  | Kerebih H et al [2017] | Y | Y | Y | Y | Y | Y | Y | Y | N | Y | 9 | Low risk |
|  | Menberu M et al [2018] | Y | Y | Y | Y | N | Y | N | Y | Y | Y | 8 | Low risk |
|  | Rathod SD et al [2016] | Y | Y | N | Y | Y | N | Y | Y | Y | Y | 8 | Low risk |
|  | Tesfaye Y et al [2020] | Y | Y | Y | Y | Y | Y | Y | Y | N | Y | 9 | Low risk |
|  | Yeshanew B et al [2019] | Y | Y | Y | Y | N | Y | Y | Y | Y | Y | 8 | Low risk |
|  | Alem A et al [1999] | Y | Y | N | Y | Y | Y | N | Y | Y | Y | 8 | Low risk |
|  | Girma E et al [2011] | Y | Y | N | Y | Y | Y | N | Y | Y | Y | 8 | Low risk |
|  | Azale T et al [2016] | Y | Y | N | Y | Y | Y | Y | Y | Y | Y | 9 | Low risk |
|  | Shumet S et al[2021] | N | Y | Y | Y | Y | N | Y | Y | Y | Y | 8 | Low risk |
|  | Negash A et a(2020] | Y | Y | N | Y | Y | Y | N | Y | Y | Y | 8 | Low risk |
|  | Teshager S et al[2020] | Y | Y | N | Y | Y | Y | Y | N | Y | Y | 8 | Low risk |
|  | Getaneh E et al[2021] | Y | Y | N | Y | Y | Y | N | Y | Y | Y | 8 | Low risk |

Note: Y, Yes; N, No; Q1, Representatives of the target population; Q2, Representativeness of the sampling frame; Q3, Random sampling or census; Q4, Minimal response bias; Q5, Data were collected directly; Q6, Acceptable case definition used in the study; Q7, Valid and reliable measurement; Q8, The same mode of data collection for all study subject; Q9, Appropriate length of prevalence period for parameter of interest and Q10, Appropriate numerators and denominators of interest.
